# ISCEV and IPS guideline for the full-field stimulus test (FST)

**DOI:** 10.1007/s10633-023-09962-7

**Published:** 2024-01-18

**Authors:** J. K. Jolly, J. R. Grigg, A. M. McKendrick, K. Fujinami, A. V. Cideciyan, D. A. Thompson, C. Matsumoto, R. Asaoka, C. Johnson, M. W. Dul, P. H. Artes, A. G. Robson

**Affiliations:** 1 Vision and Eye Research Institute, Anglia Ruskin University, Young Street, Cambridge, CB1 2LZ UK; 2 Save Sight Institute, Specialty of Clinical Ophthalmology and Eye Health, Faculty of Medicine and Health, The University of Sydney, Sydney, NSW Australia; 3 Eye Genetics Research Unit, Sydney Children’s Hospitals Network, Save Sight Institute, Children’s Medical Research Institute, The University of Sydney, Sydney, NSW Australia; 4 Lions Eye Institute, University of Western Australia, Perth, Australia; 5 School of Allied Health, University of Western Australia, Crawley, Australia; 6 Laboratory of Visual Physiology, National Institute of Sensory Organs, National Hospital Organization Tokyo Medical Center, Tokyo, Japan; 7 Institute of Ophthalmology, University College London, London, UK; 8 Center for Hereditary Retinal Degenerations, Scheie Eye Institute, University of Pennsylvania, Philadelphia, USA; 9 The Tony Kriss Visual Electrophysiology Unit, Clinical and Academic, Department of Ophthalmology, Sight and Sound Centre, Great Ormond Street Hospital for Children NHS Trust, London, UK; 10 Great Ormond Street Institute of Child Health, University College London, London, UK; 11 Department of Ophthalmology, Kindai University, Osakasayama, Japan; 12 Department of Ophthalmology, Seirei Hamamatsu General Hospital, Hamamatsu, Shizuoka Japan; 13 Seirei Christopher University, Hamamatsu, Shizuoka Japan; 14 Nanovision Research Division, Research Institute of Electronics, Shizuoka University, Shizuoka, Japan; 15 The Graduate School for the Creation of New Photonics Industries, Shizuoka, Japan; 16 Department of Ophthalmology and Visual Sciences, University of Iowa, Iowa City, Iowa, USA; 17 School of Optometry, The Ohio State University, Columbus, IA USA; 18 Department of Biological and Vision Science, College of Optometry, State University of New York, New York, USA; 19 Faculty of Health, University of Plymouth, Plymouth, UK; 20 Department of Electrophysiology, Moorfields Eye Hospital, London, UK

**Keywords:** Threshold, Sensitivity, Scotopic, Photopic, Clinical standards, International Society for Clinical Electrophysiology of Vision, Imaging and Perimetry Society

## Abstract

The full-field stimulus test (FST) is a psychophysical technique designed for the measurement of visual function in low vision. The method involves the use of a ganzfeld stimulator, as used in routine full-field electroretinography, to deliver full-field flashes of light. This guideline was developed jointly by the International Society for Clinical Electrophysiology of Vision (ISCEV) and Imaging and Perimetry Society (IPS) in order to provide technical information, promote consistency of testing and reporting, and encourage convergence of methods for FST. It is intended to aid practitioners and guide the formulation of FST protocols, with a view to future standardisation.

**Supplementary Information:**

The online version contains supplementary material available at 10.1007/s10633-023-09962-7.

## Introduction

The full-field stimulus test (FST) was developed by Roman and colleagues for the assessment of vision in patients with severe vision loss. Roman et al. 2005 [1] used a modified perimeter to introduce the concept of perception with full-field flash stimuli using manufacturer’s software. Subsequently, Roman and colleagues used a ganzfeld stimulator, as used for full-field electroretinography (ERG), to extend the dynamic range of available stimuli, with custom software to drive the system [2]. For a pre-defined stimulus and adaptation state, FST is used to provide a measure of visual function originating from any location in the retina and is presumed to originate from the most sensitive photoreceptors [1, 3–5].

The technique has been used increasingly in clinical trials for novel therapies, particularly gene therapy, to assess the restoration or preservation of retinal function [6]. FST is especially useful for detecting residual vision in patients with severe vision loss, including those with undetectable or severely abnormal full-field ERGs, or when poor fixation or nystagmus makes visual field tests difficult or impossible to perform.

The International Society for Clinical Electrophysiology of Vision (ISCEV, www.iscev.org) publishes standards, guidelines and extended protocols for electrophysiological methods including the full-field ERG [7–21]; the Imaging and Perimetry Society (IPS, www.perimetry.org) publishes guidelines for psychophysical tests such as perimetry [22]. The FST and full-field ERG require diffuse flashes of light and are frequently performed using the same ganzfeld flash stimulator. This document is a collaboration between the ISCEV and the IPS and is a guideline for FST testing, informed by methods that have been published and graded for quality. It is intended to aid practitioners and guide the formulation of FST protocols, to promote conformity and to facilitate meaningful inter-laboratory and inter-study comparisons, with a view to future standardization of routinely used FST methods.

It is highlighted that the term “FST” has been given a number of definitions historically in the literature and that this guideline defines FST as full-field stimulus test. Online Appendix 1 provides definitions of other relevant terminology.

## Scope and applications

The FST has value to test and monitor disease progression and/or treatment efficacy of therapeutic interventions, notably in natural history studies of retinal dystrophies, or in clinical trials aimed at arresting retinal degeneration or restoring retinal function [23]. An advantage over more traditional methods is that relatively small decreases or improvements in retinal function may be established, reflecting activity driven by the most sensitive retinal photoreceptors [24]. In contrast, methods such as full-field ERG normally depend on the response of millions of photoreceptors to suprathreshold stimuli, and small changes in retinal function may be undetected, especially in the presence of severe global retinal dysfunction.

Adult patients and older children may undertake FST, likely as potential candidates for clinical trials aimed at monitoring or restoring retinal function, or to monitor safety of therapeutic interventions. Age is an important consideration and may preclude or limit applicability (Sect. "Reduced protocol").

## Technical considerations

In order to inform this protocol data were sourced from recent reviews and a systematic review (Online Appendix 2A) (PROSPERO ID 453200) available on https://www.crd.york.ac.uk/PROSPERO/, in which papers were graded for quality according to the methodology of a modified Newcastle grading protocol shown in Online Appendix 2B [25]. The papers included in the final review are listed in Table 2 [1, 2, 5, 24, 26–48]. For further details see Online Appendix 3.

The FST is a psychophysical testing method that uses a range of physically well-defined light stimuli to determine visual detection thresholds, i.e. the flash strength corresponding to the stimulus being seen approximately half the time. The spectral and temporal properties of the stimulus, the algorithm used to present sequential stimuli, audible cues, and response choices can contribute strongly to the resulting FST threshold. These and other considerations are outlined below.

### Stimulus parameters

#### Colour

The simplest form of FST uses a white (achromatic) stimulus which provides no information to differentiate between light sensitivity originating from rods or cones or both. More commonly, two or more spectrally distinct short- and long-wavelength stimuli (blue and red) are used to obtain information regarding different photoreceptor types. Other colours such as green (513 nm) have been used rarely [49]. A white stimulus is produced by using a broadband white light with a colour temperature of 6500 K [29, 31, 36]. Short wavelength (blue) and long wavelength (including red) stimuli can be produced using a range of LEDs [1, 2, 5, 27, 30, 34, 35, 50]. Short wavelength LEDs with wavelengths between 444 and 470 nm and longer wavelength LEDs with wavelengths between 538 and 670 nm have been used.

#### Temporal stimulus characteristics

##### Stimulus duration and temporal envelope

Stimulus duration (presentation time) is the time between stimulus onset and offset. For clinical visual psychophysical testing, presentation times are usually selected to exceed a critical duration (defined by Bloch’s law) of around 100 ms, which defines the stimulus duration beyond which temporal summation no longer influences threshold [51]. In classic (standard automated) perimetry, stimulus durations are usually between 100 ms (Octopus perimeters, Haag-Streit) and 200 ms (Humphrey Field Analyser, Carl Zeiss Meditec), which also is shorter than the typical time taken to execute a saccadic eye movement. With a few exceptions (frequency-doubling technology, flicker perimetry) the temporal envelope of stimuli in perimetry is assumed to be a square-wave [52]. Classic brief duration visual stimuli were produced with a shutter having a sudden onset and offset. Modern FST stimuli are produced with LEDs driven with pulse-width modulation where the current is cycled on and off faster than visual perception. The duration of the LED light stimulus defines both the stimulus strength as well as the duration. Stimulus durations for FSTs reported in the literature range from short variable duration ≤ 4 ms stimuli to longer fixed duration stimuli of 200 ms [2, 29]. Longer stimulus durations allow for greater disease severities to be measured by extending the range to stronger lights, whereas shorter stimuli allow measurement close to normal dark-adapted absolute thresholds by extending the range to dimmer lights.

##### Interstimulus interval

The interstimulus interval (ISI) is the time between the onset of successive stimuli. In conventional perimetry, it is typically around 1400 ms. Many modern thresholding tests use adaptive timing in which the pace of the test is interactively adapted to the speed of the patients’ responses [52], providing for a faster and more engaging test. In the context of FST, particularly when dark-adapted, a longer ISI would minimise the influences of sequential stimulus presentations on threshold due to changes in adaptation state. Typically, the FST uses an ISI of less than 2.5 s but as long as 5 s has been reported [30, 39]. However, there is a difference when using a one button versus a two button response system (see Sect. "Response button"). When two buttons are used, the time stops when either button is pressed so the full ISI time window is rarely used. In a system with only one button, the ISI time will be utilised in full when no stimulus is seen and no button pressed, so in this scenario it is important to have the shorter period to avoid unnecessary prolongation of the test.

##### Response window

The response window is the time, relative to stimulus onset, during which responses are accepted as “valid”. This window is typically around 800 to 1000 ms for single button testing. Responses that occur implausibly early, e.g.  < 100 ms after stimulus onset, may be rejected as likely false positive responses [52]. However, it can be difficult to distinguish “anticipatory” false-positive responses from delayed responses to a previous stimulus for single button testing paradigm. The response window is more flexible for two button yes–no testing paradigm.

### Clinical protocol

#### Pupillary dilation

The FST is a measure of maximum sensitivity which depends on the retinal illuminance, the latter being directly proportional to pupil area (Online Appendix 1). FST is thought to require mydriasis (pupil dilation) to help standardise retinal illumination. Mydriasis may reduce inter-subject and inter-session variability, which is of particular relevance in monitoring studies, and has been specified in most of the major studies [1, 2, 5, 26, 27, 29–31, 34–38, 47, 48, 50].

#### Adaptation

FST can be conducted in both light and/or dark-adapted conditions. Both conditions must be controlled, prior to the start of testing, as the baseline level of retinal light or dark adaptation can influence final thresholds and the test outcome.

Widely different periods of adaptation have been used, ranging from no adaptation to 2 h, [1, 2, 26, 27, 29–32, 34, 36–40, 47, 48, 50] with a median time of approximately 45 min for dark-adapted testing. In disorders characterised by predominant rod dysfunction where dark adaptation may be impaired or delayed, a longer fixed period of dark adaptation may be needed to enable measurement of maximum sensitivity [53]. The second eye tested usually undergoes a longer period of dark adaptation compared to the first eye. Optimising the dark adaptation period for the disease process or considering randomising the order of testing should inform interpretation.

#### Auditory signal

The use of auditory signals to enhance responses to psychophysical testing has been reported in the perimetry literature [54, 55]. During FST, an auditory prompt signals to the subject when they should respond “yes” (seen) or “no” (not seen) for two button testing paradigm [5, 24, 26, 27, 29, 31, 33, 34, 36, 41–43]. An auditory signal is generally not relevant for one button testing paradigm [32].

#### Fixation

As the FST is a global response, fixation is not considered important and is rarely reported.

#### Learning effect

Typically, in psychophysical testing, there is a significant learning effect as individuals adjust to the testing procedure. This has been investigated extensively in perimetry with recommendations to disregard the first three test results in Humphrey field testing [56] and first test for fundus-tracked perimetry [57]. Learning effect has not been formally investigated in FST.

#### Test–retest variability

Knowledge of test–retest variability is essential to define a significant change following treatment. Roman et al. reported intervisit repeatability of 0.39 log for inherited retinal degeneration patients [1]. Dimopolous et al. reported an intervisit repeatability of 0.27 log and 0.23 log for blue and red stimuli, respectively [5]. Similar test–retest values have been reported for control and patient groups, as well as for all colours. Repeatability should reflect the units of measurement such as log10 units for thresholds measured in log10 units.

#### Repeat testing

Common practice is to conduct multiple assessments and take the average as the final result. Based on the within-subject standard deviation for white (achromatic stimuli), it has been estimated that four repeats are optimal [1]. A power calculation using a 2-sample, 2-sided *t* test to detect FST changes of 5 dB or more between the means of two sessions using 6 samples each would yield a power of 98% at a significance level of 5% [32]. However, it is important to avoid extended test sessions that may affect results due to subject fatigue. The majority of studies reviewed took an average of 3 measurements [5, 26, 27, 29, 36, 44–46, 50].

### Psychometric function and thresholding paradigms

Historically, there have been various methods of performing measurements of visual threshold: (A) the method of adjustment, where the observer varies the strength of a stimulus until it is just detectable, (B) the method of limits, where the observer determines the transition from seeing to non-seeing or vice versa by observing a sequence of ascending or descending light steps until the transition point is reached, (C) the method of constant stimuli in which a series of presentations are provided that are assumed to be above and below the presumed threshold to define a frequency of seeing curve that provides detection performance, and (D) a staircase procedure, in which the stimulus strength is increased and decreased to determine a series of reversals (seeing to non-seeing and vice versa) to identify average detection sensitivity. Each of these procedures has certain advantages and limitations, and the amount of time to perform the measurements varies considerably [58].

The most comprehensive description of stimulus–response relationships in psychophysical data is the psychometric function. This sigmoidal (s-shaped) function describes visual performance (e.g. % of seen) over a wide range of stimulus strengths. Weak stimuli are associated with a low probability of being seen and strong stimuli with a high response probability. Classically, psychometric functions are measured through the “method of constant stimuli”, in which a stimulus is repeatedly shown in order to establish how often it is seen at each stimulus level tested, according to a pre-determined probability criterion.

For most clinical applications, extreme performance levels (close to 0 or 100%) may not be greatly informative. In these situations, a single point estimate of threshold (or sensitivity) may be a more parsimonious descriptor of performance. The term “threshold” refers to the stimulus strength associated with a particular level of performance, typically 50%, but other values are possible. Sensitivity is the inverse of threshold, but both terms tend to be used interchangeably in the clinical literature.

Thresholds can be derived by statistical modelling (probit, maximum-likelihood, etc.) from frequency-of-seeing type data, or more directly through psychophysical techniques such as staircase, bracketing, or adaptive procedures which depend on the responses during testing [59]. Bayesian thresholding techniques (e.g. Zippy Estimation for Sequential Testing, ZEST) can be constructed to use “prior information” (e.g. the distribution of thresholds in a population), yielding greater efficiency.

The strategies chosen for FST balance speed and accessibility. A majority of studies report a 4–2 staircase thresholding [1, 2, 24, 29, 31, 35, 42, 43], though the number of reversals or steps to final thresholding vary in the literature. Those that report the methodology in the literature may be unaware of commercial proprietary implementation of the thresholding algorithm and may be reporting the previously published method of a 4–2 staircase which may not the method implemented in proprietary software. Perimetry has seen an evolution of thresholding algorithms and FST methods are also changing. Current methods include an abbreviated method of constant stimuli with pseudo-random expansion to detect the endpoints of the range along the slope of the psychometric function. Some manufacturers report using an 8–4-2–1 threshold staircase. As with perimetry, careful patient instruction will increase reliability [32].

**Fig. 1:**
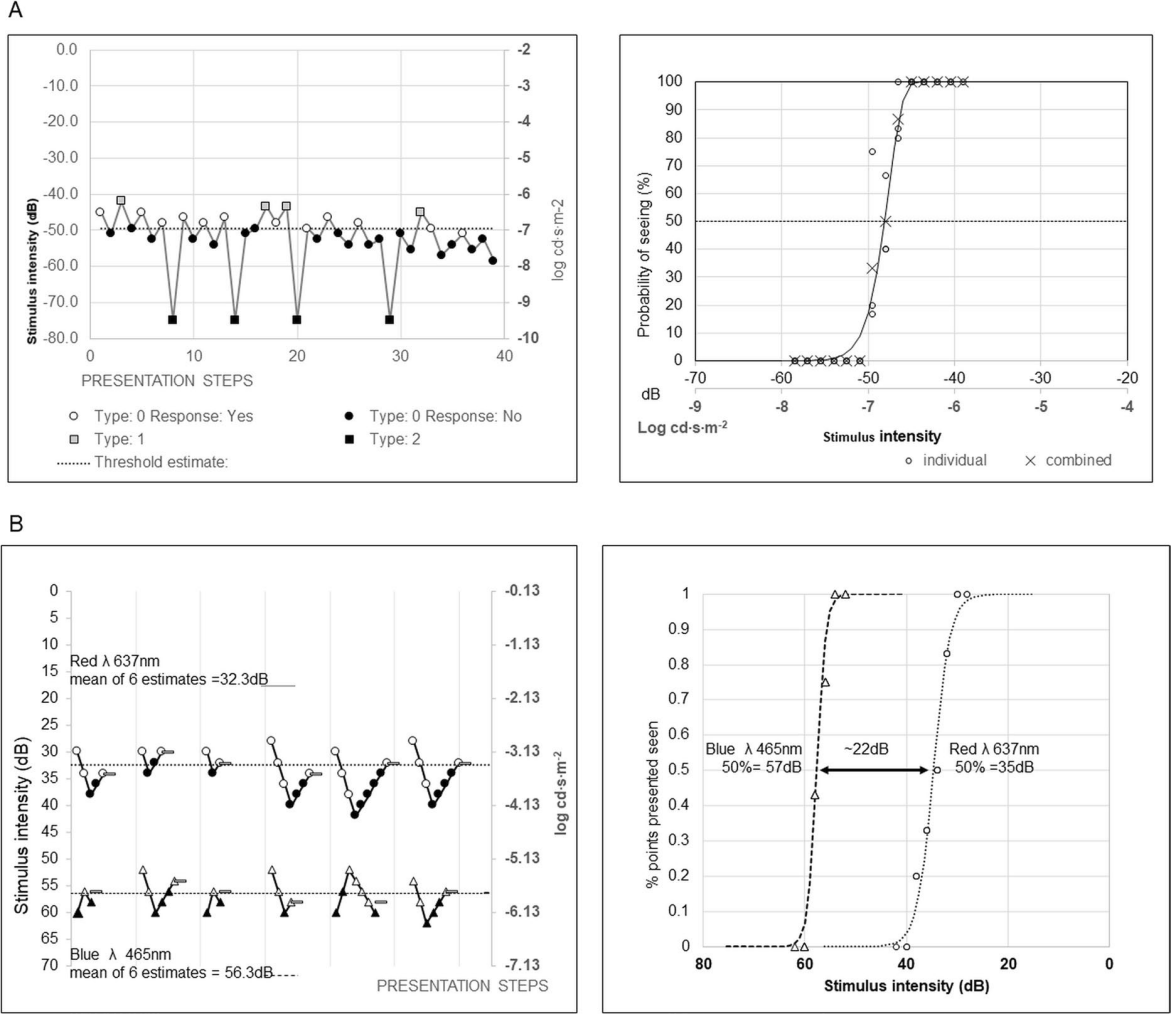
Examples of seen–unseen two button responses with corresponding modified Weibull function fitted to the data. (**A**) Top panel shows data collection following 45-min dark adaptation to a white (6500 k) stimulus with a variable duration of less than 4 ms presented in a ganzfeld (reference 0 dB = 0.01 cd∙s∙m^−2^). A proprietary presentation order of stimuli is demonstrated on the left panel, in this instance with 8 catch trials. A two button response indicates whether the stimulus is seen or unseen within a 5 s response window after an audible cue. The right panel shows probability or percentage of seen stimuli plotted against stimulus strength (“intensity”) and fitted by a modified Weibull function to the data combined from individual trials, which provides a 50% seeing mean estimate of 48 dB [− 6.8 log cd∙s∙m^−2^]. (B)The bottom panel displays six consecutive measurements using 4–2 staircase method with 2 reversals in each case. Blue (465 nm) and red (637 nm) flash stimuli with a fixed duration of 200 ms were presented following 45-min dark adaptation in a ganzfeld stimulator (reference 0 dB = 3.7 cd∙m^−2^). A single button press indicates seen. Plotted stimuli seen are indicated by open symbols, not seen by filled symbols. Triangles and circles for blue and red stimuli, respectively. The stimulus strength of the last seen is taken as the threshold, marked with a horizontal line. This process is repeated 6 times for each stimulus and an average of thresholds and standard deviation taken. The corresponding plotted threshold and modified Weibull functions are shown in the panel on the right, to provide an alternative, but similar, estimates of the 50% seen thresholds of 57 dB blue and 35 dB red, with a blue-red difference of 22 dB

The most commonly used function for the FST is the two-parameter modified Weibull fit, estimating the stimulus strength associated with 50% probability of detection based on staircase responses (Fig. 1) [5, 26–30, 33, 34, 36–40, 44–48, 50]. The equation of the Weibull function utilised is specified below, though all details of the modifications are not in the public domain and remain proprietary.

$$P\left( {seen} \right) = 1 - e^{{ - \left( {\frac{strength}{{threshold}}} \right)^{slope} }}$$

where *P* = probability, *e* = exponential distribution, strength is measured in cd∙s∙m^−2^, threshold measured in cd∙s∙m^−2^.

#### Response button

FST can be performed with a single button response where the patient reports only seen stimuli, or two button response where the patient is typically prompted with an audible cue and reports whether the stimulus is seen or not seen by pressing one of two buttons [32]. In general, single button response offers the advantage of simplicity and patients may be more familiar with the process from previous perimetric testing. Two button testing allows for quantification of both seen and unseen response times, of potential value in quality of data metrics.

### Analysis

In visual psychophysics, patient responses reflect not only the underlying sensory capability but also extra-sensory influences such as response criteria, attentiveness, and fatigue [60].

Historically, the patients’ response criteria are assessed by interspersing a small number of catch trials in the stimulus sequence. For example, responses to blank trials during which no stimulus was present are interpreted as false positives and may suggest either the patient has a low criterion for responding or experiences photopsias. False positives may also be logged when the patient provides a delayed response to a preceding stimulus that is implausibly early after the next stimulus. False negatives are assessed by the failure to respond to strong stimuli previously seen [29]. False negatives require an estimate of the threshold and may have limited utility in patients with severe vision loss approaching the limits of the equipment.

The reproducibility of test results can be assessed in terms of test–retest variability via Bland–Altman analyses [61]. A potential limitation arises when variability is markedly non-uniform across the dynamic range of the technique (heteroscedasticity, as in clinical perimetry). In these situations, empirical test–retest intervals can be estimated as an alternative.

## Protocol specification

Recommendations are based on a combination of best practice theory and published protocol parameter derived from the systematic review.

### Stimulus parameters

#### Colour

This guideline specifies the use of LEDs with centre wavelengths for the short wavelength (blue) stimulus of between 444 and 470 nm and for long wavelengths (red) between 620 and 670 nm. Once tested, the difference between the red and blue responses should be calculated (see Sect. "Photoreceptor mediation"). White colour temperature should be 6500 K.

#### Auditory signal

An auditory cue is recommended for the two button seen–not seen testing paradigm; it is not necessary for single button testing. It is acknowledged that auditory cues may not be appropriate in patients with severe hearing loss and tactile cues may have to be used.

#### Stimulus duration and interstimulus interval

The stimulus duration must be defined. Literature to date has reported either brief (≤ 4 ms) or longer duration (200 ms) stimuli. Interstimulus interval must be defined. Previous studies have specified an interstimulus interval of approximately 1 s for single button paradigms, or up to 5 s for two button paradigms [29, 47].

Break periods should be provided between bouts of testing to allow re-adaptation to the dark and to reduce fatigue. The length of these should be defined, ideally > 3 min.

### Clinical protocol

#### Dilation

Pupil dilation is a requirement of this guideline for clinical trials and research. Pupil diameters should be measured and recorded. If mydriasis is contraindicated, this must be acknowledged as a departure from the guideline and the likely influence on thresholds and interpretation of results considered.

#### Adaptation

For light-adapted testing, a minimum light adaptation period of 5 min is recommended. Prior to dark-adapted testing, a period of 45-min dark adaptation is recommended, subject to consideration of the patient’s diagnosis. This can be assessed by checking the dark adaptation characteristics in a sample of disease patients prior to finalising the protocol. In retinal disorders characterised by severely delayed dark adaptation, a longer fixed period of dark adaptation may be used, if necessary to obtain detectable or robust responses, although care must be taken to ensure consistency between patients and serial assessments.

#### Response button

Whether a one button or two button box is used and the manner in which it is used should be specified, such as alternative choice with an auditory cue.

#### Patching

Adequate patching of the contralateral eye to prevent light stimulus “leakage” is essential and may require use of additional patching to provide blackout conditions. It is highlighted that the sensitivity of one eye may be orders of magnitude higher than the fellow, e.g. in uniocular gene-based treatment trials. The order of eye testing should be reported as the second tested eye may have longer dark adaptation.

### Psychometric function and thresholding paradigms

#### Thresholding algorithm

The algorithm for sequential presentation of stimuli should be specified [1, 2, 24, 29, 31, 35, 42, 43]. The observer or patient should be instructed clearly to optimise compliance and reliability of measurements. FST is a psychophysical test and patient instruction has a profound impact on the reliability of testing and final threshold obtained. On this basis, an example of good practice instructions is provided in Online Appendix 4.

#### Starting stimulus

Under dark-adapted testing, longer wavelength (red) stimuli should be presented first followed by shorter wavelength (blue) stimuli, to minimise the disruption of the dark-adapted state of the retina.

It can be helpful to commence testing closer to the expected final threshold where possible, to reduce test time.

#### Threshold

Threshold should be defined as the stimulus strength associated with 50% probability of detection, based on the psychometric function, e.g. two-parameter modified Weibull fit.

### Analysis, interpretation, and reporting

#### Learning effect

Observers should perform several trial runs for training, e.g. one to two minutes, immediately prior to testing. More are recommended if needed to optimise patient comfort and compliance.

#### Catch trials

False positive catch trials should be included in the test in order to provide some indication of performance reliability during testing. False negative catch trials if recorded should be interpreted with caution as they are a suprathreshold presentation and have the potential to disrupt the dark adaptive state.

#### Test–retest variability

Data analysis requires an appreciation of test–retest variability and this should be examined for the system and stimuli being used, and for the population being studied.

#### Repeat testing

If the first three tests are consistent, then three are sufficient to comply with this guideline. In cases of high intra-subject variability, attempts should be made to encourage better compliance and further repeats obtained to optimise consistency. This applies separately to each test condition used.

#### Units of measurement

Threshold results should be reported in units of log cd·s·m^−2^ for brief stimuli, or in units of log cd·m^−2^ with longer duration stimuli together with the stimulus duration. Considering the use of chromatic stimuli under dark- and light-adapted conditions, units must be specified in photopic or scotopic units (phot-cd or scot-cd) as appropriate to the application. Decibels units (dB = 10log_10_) should not be used, as although widely employed in psychophysical techniques such as perimetry, it is a relative scale dependent on the 0 dB point (reference stimulus strength).

#### Photoreceptor mediation

In two-colour chromatic FST testing, the difference in thresholds between short- and long-wavelength tests can provide an estimate of the photoreceptors contributing to each response. The precise difference will partly depend on the radiometric properties of the LED stimuli, and the equipment used must therefore be specified [2].

#### Reference ranges

Establishing reference (“normative”) values involves recruiting and testing sufficient reference subjects per clinically relevant partition, and establishing laboratory-specific reference limits is generally considered the optimal process. If external or published reference data (e.g. [62]) are to be used they must be verified as appropriate for the local methods and equipment, with an understanding of possible limitations and how reference limits were defined.

#### Quality control

The quality and consistency of measurements should be monitored and reported. This could be in the form of monitoring the quality of the responses as they were collected and repeating if required and/or excluding unreliable or low-quality tests, although if necessary this should be clearly acknowledged. The consistency of data and shape of the psychometric function fitted to the measurements should be examined to assess the reliability of the responses (Online Appendix 5). If a disproportionate number of points are located around the 0% and 100% locations compared to intermediate values along the probability curve, this may indicate a skew in the response characteristics and impair curve fitting.

#### Reporting recommendations checklist

In order to facilitate comparison between reports, the following minimum information should be specified in all FST reports:

1. Adherence to this guidance should be stated, and any departures acknowledged and justified.
2. Equipment used and full details of test paradigm employed.
3. For proprietary software, if full details are not available, it is essential to specify the version number, settings and protocol.
4. Stimulus duration and interstimulus interval.
5. Stimulus colour including the peak wavelength of LEDs used.
6. Pupil sizes after mydriasis.
7. Duration of light and dark adaptation including periods of re-adaptation.
8. The quality assessments employed.
9. The number of tests completed for each stimulus condition.
10. The eye tested and order of eyes tested.
11. The response method (e.g. use of a one or two button box and test paradigm) and thresholding algorithm.
12. Thresholds provided in log cd·s·m^−2^ for brief flash durations under 4 ms, and in log cd·m^−2^ for longer durations.

## Reduced protocol

When comprehensive FST is not possible, such as for young children, those with special needs, and adults unable to comply with routine testing, a reduced protocol may be considered. Modifications may have a negative impact on reliability and sensitivity, but there remains potential to yield meaningful and clinically useful results, providing the core principles of FST are retained.

It is well recognised that paediatric psychometric testing of vision can be challenging, depending on the age, capability and compliance of the child. Maturity and compliance can vary significantly between children of the same age and across ages requiring examiner’s judgement [63]. It has been reported that children older than 8 years are able to reliably perform perimetry [64]. Published FST data suggest children over 6 years without physical or neurodevelopmental impairment may successfully complete the FST. Those under 6 years, or older children/adults unable to understand the requirements of the test, or unable to physically comply with a button press may need support or adaptations.

Any test modification may impact the reliability and sensitivity of a test and adaptations must be documented carefully so that the test protocol can be replicated for monitoring. Compliance must be detailed and recorded to inform data interpretation. At this time, there are no evidence-based age-related modifications for children and this is an area that requires further research. This guidance is based on guidance from other psychophysical testing.

Shortened protocols will usually involve changing at least one of several parameters. Ways of making the test into a game, involving the child’s carer or an older sibling, and allowing breaks for drinks and snacks between tests may be helpful. The period of dark adaptation may be shortened, or testing performed without mydriasis. There may be fewer types of stimuli per test session such as prioritising white over colour stimuli, depending upon the clinical priority, with shorter but more frequent test sessions. Simultaneous rather than sequential testing of both eyes and simpler response protocols may be considered, e.g. using one button rather than two or allowing verbal or tactile responses that a carer reports back. This may require a longer ISI to provide time for the feedback and the carer must be counselled not to bias responses. Balancing quality control measures, such as decreasing the number of catch trials, with test duration is also important.

### Supplementary Information

Below is the link to the electronic supplementary material.

Supplementary file1 (PDF 583 kb)
