# Supplementary material for: ISCEV and IPS guideline for the full-field stimulus test (FST)
Source: Doc Ophthalmol. 2024 Jan 18;148(1):3–14. doi: 10.1007/s10633-023-09962-7 (PMC10879267; doi:10.1007/s10633-023-09962-7)
Supplement: Supplementary file 1 — Supplementary file1 (PDF 583 kb) [file 10633_2023_9962_MOESM1_ESM.pdf]

Appendix 1: Definition of terms

This definition section provides a guide to the terminology that is used in the commercially available systems. Terminology varies between systems in ways that tend to reflect their origins and development from either an electrophysiological based system or a perimetry based system.

Full Field Stimulus Test (FST) aims to measure lowest luminance flash which elicits a visual sensation perceived by the subject. The FST evolved from dark adapted perimetry. In conventional perimetry, the standard unit of measurement for the visual field is the differential light sensitivity (DLS). This is defined as the threshold of perception of a test object, relative to its background. The subjectively perceived brightness of an object such as the FST stimulus are related to their luminance.

To understand the quantification of the test object various photometry terms are required.

Luminous flux (ϕ): is the power of visible light emitted by an object and is measured in the International System of Units (SI) unit Lumens (lm). Luminous flux takes into account the entire range of wavelengths that are visible to the human eye. The term describes the brightness of a light source.
Luminous intensity (I): is the luminous flux emitted in a particular direction. $I= \phi/ \Omega$ where Ω is the solid angle expressed in steradians Luminous intensity is measured in candela (cd).
Illuminance (E): is the luminous intensity per unit of area of an object. $E=I/A$ where A is the reflective area. Unit is the lux ($lm \cdot m^{-2}$). Illuminance is a measure of the amount of light falling on a surface from a light source. It is measured with a luxmeter.
Luminance: the amount of light emitted or reflected from a surface in a particular direction. It is defined as the amount of luminous flux per unit area, per unit solid angle, in a specific direction. Luminance is measured in candelas per square meter ($cd \cdot m^{-2}$). Luminance refers to the brightness of a surface e.g., of long duration or background light.
Stimulus flash strength: is measured in luminance*time ($cd \cdot m^{-2}.s$). This is equivalent to candela-seconds per square meter ($cd \cdot s \cdot m^{-2}$). This is a measurement of luminous power (flux) per unit solid angle (steradian) per unit surface area.

SI Units Defined and compared to older or non-metric terms

Nits: are a measure of luminance. 1 nit = 1 candela per square meter.
Lux (lx): is a unit of measurement for illuminance. It is defined as the amount of light that falls on a surface that is one square meter in size, and that is located at a distance of one meter from a light source with a luminous intensity of one candela.
Steradian (sr) is a unit of measurement for solid angles in three-dimensional space. It is defined as the solid angle that subtends a surface area of one square meter at the centre of a sphere with a radius of one meter.
Troland (Td) is a unit of measurement of retinal illuminance, the amount of light reaching the retina of the eye. Specifically, a troland is defined as the retinal illuminance produced by a luminance of one candela per square meter ($cd \cdot m^{-2}$) viewed through a pupil with an area of one square millimetre (mm^2).

Appendix 2: Details of systematic review

Appendix 2A: PRISMA diagram providing details of the systematic review process

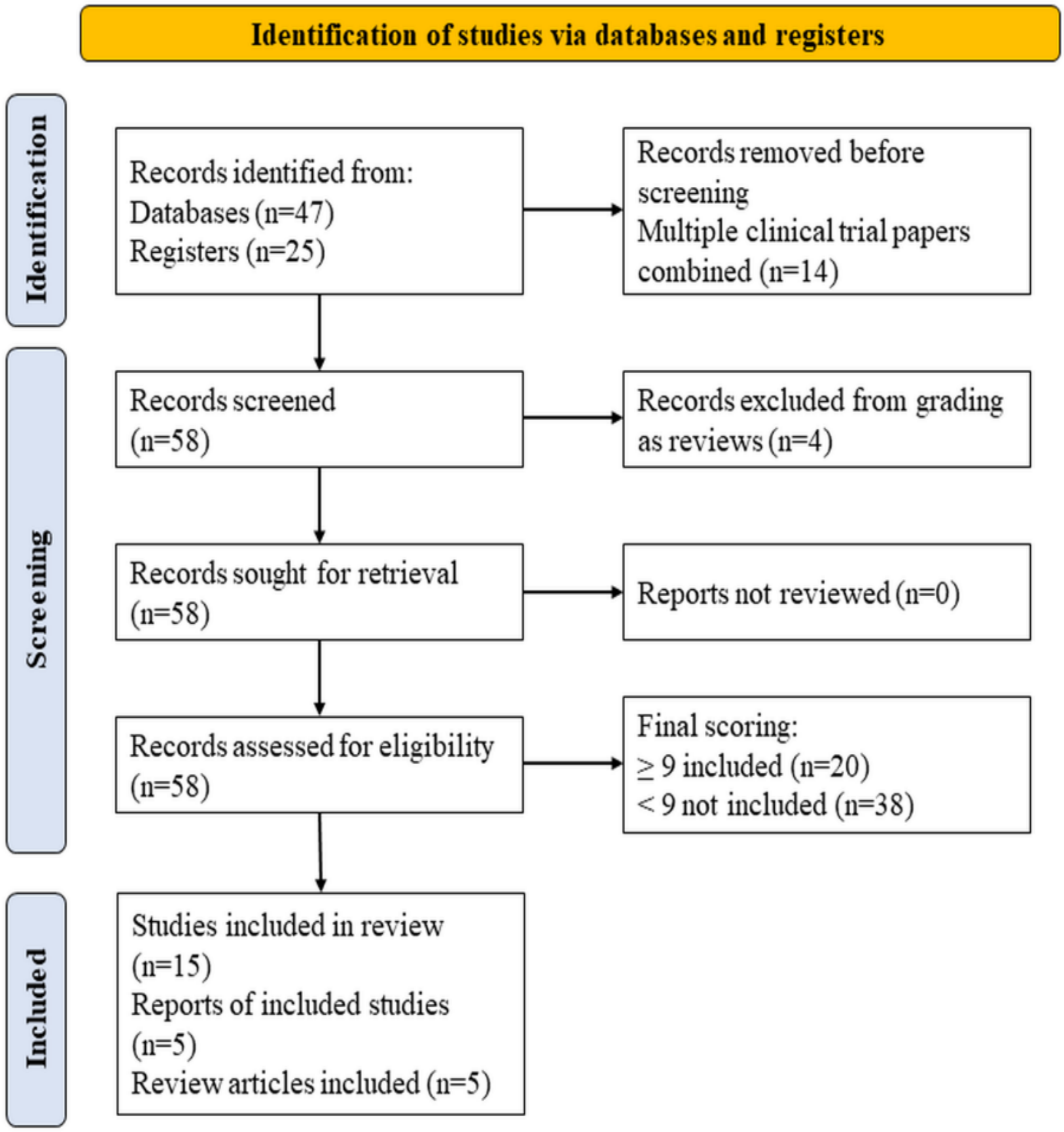

Appendix 2B: Amended Newcastle scoring tool for quality grading of the papers

**Appendix 2B: Questionnaire used to score each paper**			
	**Syntheses that will be assessed for risk of bias**	**For each study, consider what criterion is met. Each section can be scored with a maximum of one star. If the study meets at least one of the starred criteria, then place a 1 in the next column. If the study does not meet any starred criteria, then leave the next column blank.**	**Scoring**
**1**	Equipment used	a) Study identifies equipment used plus the button box *	
		b) Study identifies equipment but not full information about the set up *	
		c) No information given about equipment used	
**2**	Staircase Paradigm	a) Details of the staircase and psychometric function are given *	
		b) Only partial details given, so calculations could not be replicated	
		c) No information given about the staircase paradigm	
**3**	Stimulus colour	a) Wavelengths of all test stimuli are provided, along with details of generating sources *	
		b) Only colour names are given	
		c) No information given about stimulus colour	
**4**	Interstimulus interval	a) ISI details are given and remain stable for all recordings, unless specified. If ISI intervals varied at all, this was adjusted for in the analysis *	
		b) Not enough information about ISI given	
**5**	Inter-trial duration	a) Details given about rest periods between repeat testing of same stimuli, and of different stimuli *	
		b) Limited details given	
		c) No information given	
**6**	Eye and test order	a) Information given about the order in which the eyes were tested, and the order in which colours were tested, with full details about dark adaptation periods in between testing *	
		b) Limited details given	
		c) No information given	
**7**	Dilation status	a) Details given about dilation status before testing, and pupil diameter is recorded *	
		b) Details given about dilation status before testing, but pupil diameter not recorded *	
		c) No information given	
**8**	Length of dark adaptation	a) Information is given about length of dark adaptation prior to testing, AND re-adaptation at any points during testing *	
		b) Information is given about length of dark adaptation prior to testing, but no information given about re-adaptation periods during testing *	
		c) No information given about dark adaptation prior to testing	
**9**	Definition of 0dB point	a) Units are reported in log cd·m^{-2} or in log cd·s·m^{-2} *	
		b) Units are reported in decibels with a defined 0dB point in the above units *	
		c) Units are reported in decibels with no defined 0dB point	
		d) Units are not defined	
**10**	Number of repeats	a) Information is given on number and order of repeats, with full details of breaks and adaptation periods between repetitions given *	

		b) Information is given on number and order of repeats, but no information given on breaks and adaptation periods between repetitions *	
		c) Information given on the number of repeats only	
		d) No information given	
**11**	If audible cues used	a) Information is given about whether audible cues were used on stimulus presentation *	
		b) No information given	
**12**	Quality assessment: was any quality assessment of patient performance carried out?	a) QA was carried out and full description was given of procedure; QA results were accounted for in the analysis *	
		b) QA carried out but not accounted for in analysis, or QA not done	
		c) No mention of whether QA was done	
**13**	Whether binocular/monocular	a) Information is given about whether they tested one or two eyes *	
		b) No information given	
**14**	If the case definition or cohort inclusion criteria are adequate	a) The study population is well defined and verifiable through validated records or healthcare records *	
		b) Cases are based on participant self-reporting only	
		c) Inadequate description given	
**15**	Does the study report age of the participants?	a) Yes *	
		b) No	
**16**	Does the study report sex of the participants?	a) Yes *	
		b) No	
**17**	Does the study report ethnicity of the participants?	a) Yes *	
		b) No	

Appendix 3: Summary of High-Quality Papers scored ≥9

**Table 2: Key parameters reported used in all the high quality papers and studies.**		
Study	***Papers***	***Key Test Parameters Reported***<br>***Colour***<br>***Stimulus Duration***<br>***Inter Stimulus duration (ISI)***<br>***Units***<br>***Response Window***<br>***Dilation***<br>***Psychometric Function***<br>***Dark adaptation time***<br>***Quality Control***
Individual Studies	Collison et al. 2014[26]	Colour: White 6500K, red, and blue<br>Dilation: Yes<br>Dark adaptation: 30 mins<br>Light adaptation: 5 mins bleach for cone<br>Response: 2 buttons, audible cue<br>Psychometric Function: Diagnosys LLC Weibull Function<br>Units: 0 dB = 0.1 cd·s·m^2<br>Repeats: 3
	Collison et al. 2015 [27]	Colour: White 6500K, "Dim" LEDs, below 0.01 cd·s·m^2, 468nm (blue) and 632nm (red)<br>"Bright" LEDs, above 0.01 log cd·s·m^{-2}, 444nm (blue) and 632nm (red)<br>Dilation: Yes<br>Dark adaptation time: 30 mins<br>Response: 2 button box<br>Psychometric Function: Diagnosys LLC Weibull Function<br>Repeats: 3
	Dimopoulos et al. 2018 [5]	Colour: 456-460nm (blue), 635-538nm (red)<br>Dilation: Yes<br>Dark adaptation time: 30 mins plus 5 mins bleach<br>Response: 2 buttons forced choice<br>Psychometric Function: 4-2 staircase. Weibull function.<br>Repeats: 3<br>Units: 0 dB = 0.01 log cd·s·m^{-2}
	Jacobson et al. 2011 [28]	Colour: White, Red, Blue<br>Stimulus Duration: 200 ms<br>Psychometric Function: 4-2 staircase with 2 reversals
	Klein and Birch 2009 [29]	Colour: White (6500 K)<br>Stimulus Duration: Diagnosys LLC ≤ 4 ms; FST2 200 ms<br>ISI: 5 s<br>Dilation: Yes<br>Dark adaptation time: 30 mins<br>Response: Diagnosys 2 button box forced choice with audible cue, FST2 single button box

		Psychometric Function: 4-2 staircase. A two parameter Weibull Function. Tester defined starting value.<br>Units: 10dB = 1 log unit. Diagnosys LLC 0 dB = 0.1 log cd·s·m^{-2} FST2 0 dB = 0.74 log cd·s·m^{-2}
	McAnany et al. 2020 [30]	Colour: 642nm (red), 456nm (blue)<br>Stimulus Duration: ≤ 4ms<br>Dilation: Yes<br>Dark adaptation time: 30 mins<br>Psychometric Function: Diagnosys LLC Weibull Function<br>Repeats: 2<br>Units: 0 dB = 0.01 log cd·s·m^{-2}
	Messias et al. 2013 [31]	Colour: White (6500 K)<br>Stimulus Duration: ≤ 4 ms<br>Dilation: Yes<br>Dark adaptation time: 30 mins<br>Response: 2 button, forced choice, auditory cue<br>Psychometric Function: 4-2 staircase. Weibull Function.<br>Units: 0 dB = 0.01 log cd·s·m^{-2}
	Roman et al. 2005 [1]	Colour: White, 650nm (red), 500nm (blue)<br>Stimulus Duration: 200 ms<br>Dilation: Yes<br>Dark adaptation time: more than 45 mins<br>Psychometric Function: 4-2 staircase. Threshold based on last seen.<br>Units: 0 dB = 3.7 log cd·s·m^{-2}
	Roman et al. 2007 [2]	Colour: 637nm (red), 465nm (blue), 517nm (green), white<br>Stimulus Duration: 200 ms<br>Dilation: Yes<br>Dark adaptation time: more than 45 mins<br>Psychometric Function: 4-2 staircase with 2 reversals. Practice run and customised start point.<br>Repeats: 3<br>Units: 0 dB = log cd·s·m^{-2}
	Roman et al. 2022 [32]	Colour: White, low luminance; 647 nm (red) and 500 nm (blue), higher luminance; 655 nm (red) and 455 nm (blue)<br>Stimulus Duration: 200 ms<br>Dark adaptation time: more than 45 mins<br>Psychometric Function: 4-2-2 staircase<br>Repeats: 6
	Stingl et al. 2019 [33]	Colour: Red and blue<br>Dark adaptation time: 20 mins<br>Response: one button box<br>Psychometric Function: Diagnosys LLC Weibull Function<br>Units: 0 dB set to 0.01 log cd·s·m^{-2}
	Talib et al. 2012 [50]	Colour: White, blue, read<br>Stimulus Duration: < 4 ms<br>Dilation: Yes<br>Dark adaptation time: more than 30 mins

		Psychometric Function: Diagnosys LLC Weibull Function<br>Repeats: 3<br>Units: 0 dB = 0.1 log cd·s·m^{-2}
	Testa et al. 2021 [34]	Colour: White, "Dim" LEDs, below 0.01 log cd·s·m^{-2}, 468nm (blue) and 632nm (red)<br>"Bright" LEDs, above 0.01 cd-s/m^{2}, 444nm (blue) and 632nm (red)<br>Dilation: Yes<br>Dark adaptation time: 40 mins<br>Response: 2 button forced choice<br>Psychometric Function: Diagnosys LLC Weibull Function
	William et al 2022 [35]	Colour: 635nm (red), 513nm (green), 470nm (blue)<br>Stimulus Duration: ≤ 4 ms<br>Dilation: Yes<br>Psychometric Function: Diagnosys LLC Weibull
	Zabek et al. 2022 [36]	Colour: White (6500 K)<br>Stimulus Duration: ≤ 4 ms<br>Dilation: Yes<br>Dark adaptation time: more than 30 mins<br>Response: 2 button forced choice, auditory cue<br>Psychometric Function: 4-2 staircase. Weibull Function.<br>Repeats: 3<br>Units: 0 dB = 0.1 log cd·s·m^{-2}
Argus 2 Implant –602	Humayun et al. 2012 [37]<br>Ahuja et al. 2013 [47]<br>Ahuja and Behrend 2013 [48]	Colour: White<br>Stimulus Duration: ≤ 4 ms<br>ISI: 5 s<br>Dilation: Yes<br>Dark adaptation time: 30 mins<br>Psychometric Function: Staircase with 16 reversals. Diagnosys LLC Weibull Function<br>Units: 0 dB = 3 log cd·s·m^{-2}
hMERTK - 195	Ghazi et al. 2016 [38]	Colour: White<br>Stimulus Duration: 200 ms<br>Dilation: Yes<br>Dark adaptation time: more than 45 mins<br>Psychometric Function: Diagnosys LLC Weibull Function<br>Repeats: 5
Luxturna - 389	Bennett et al. 2016 [39]<br>Maguire et al. 2019 [40]	Colour: White, red and blue<br>Dark adaptation time: 40 mins<br>Psychometric Function: Weibull Function<br>Units: log cd·s·m^{-2}
Antisense - 969	Cideciyan et al. 2019 [41]<br>Cideciyan et al. 2021 [24]<br>Cideciyan et al. 2022 [42]<br>Russell et al. 2022 [43]	Colour: Red and Blue<br>Psychometric Function: 4-2 staircase with 2 reversals<br>Response: Single button<br>Units: log cd·s·m^{-2}

<table>
<tr><td rowspan="3">RUSH2A - 078</td><td>Birch et al. 2020 [44]</td><td rowspan="3">Colour: White, red and blue
Psychometric Function: Diagnosys LLC Weibull Function
Repeats: 3
Units: 0 dB = 0.1 log cd·s·m^{-2}</td></tr>
<tr><td>Birch et al. 2022 [45]</td></tr>
<tr><td>Hufnagel et al. 2022 [46]</td></tr>
</table>

Appendix 4: Optimising observer compliance

Effective communication between the practitioner and observer will optimise psychophysical performance and compliance, and is pivotal to accurate and reliable FST measurements. It is essential to explain to observers or patients what they should expect and what is required before testing, and to offer reassurance and/or encouragement during testing. Instructions may be tailored to be appropriate for individuals or patient groups, but explanations about key features and task are usually included and presented in a similar way for each patient. An example of such an explanation is given below for a 2-button FST, although not intended to be prescriptive or suitable for all patients.

"We are now going to test the sensitivity of your vision. You will hear a number of beeps and each time there may or may not be a light presented that is visible to you. Many of the lights will be extremely dim, much more so than you are used to seeing in your typical day or during visual field tests you have done in the past, so even if you think you have seen it, please press yes. We are trying to find out the limits of your vision so you are not expected to see all the lights. If you see a light with a beep please press the button on the right, if you don't see a light with a beep please press the button on the left. Please do not be concerned about "right" or "wrong" responses, or about where the light is coming from or what colour it is. You do not need to respond too quickly, but it is equally important not to be too hesitant, and to act on your first impressions, and to press the button on the right if you think you see any light."

Appendix 5: An example of a poor quality response and problem solving

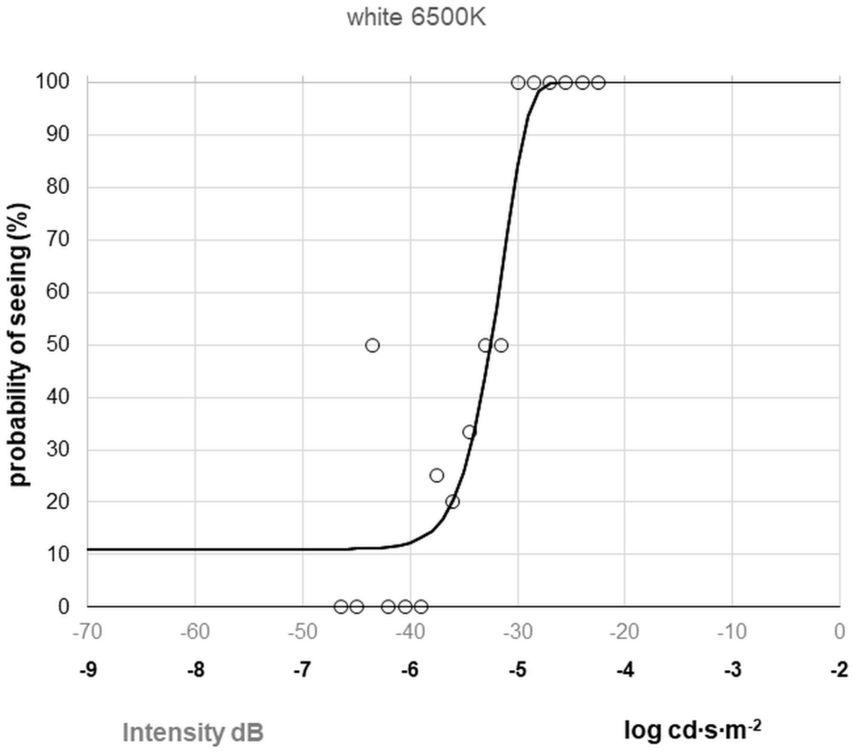

Figure: The modified Weibull function does not reach the 0% so the final output is not ideal. This may be as a result of the outlier. This could be corrected by changing the test parameters such as extending the interstimulus intervals, altering the instructions, or proving more practice.

There are many other possible errors, such as but not limited to; no evidence of a slope, skewed responses, large variability in responses, and ceiling or floor effects.
